# Supplementary material for: Interpreting pathways to discover cancer driver genes with Moonlight
Source: Nat Commun. 2020 Jan 3;11:69. doi: 10.1038/s41467-019-13803-0 (PMC6941958; doi:10.1038/s41467-019-13803-0)
Supplement: Supplementary file 1 — Supplementary Information [file 41467_2019_13803_MOESM1_ESM.pdf]

Supplementary Figure 1

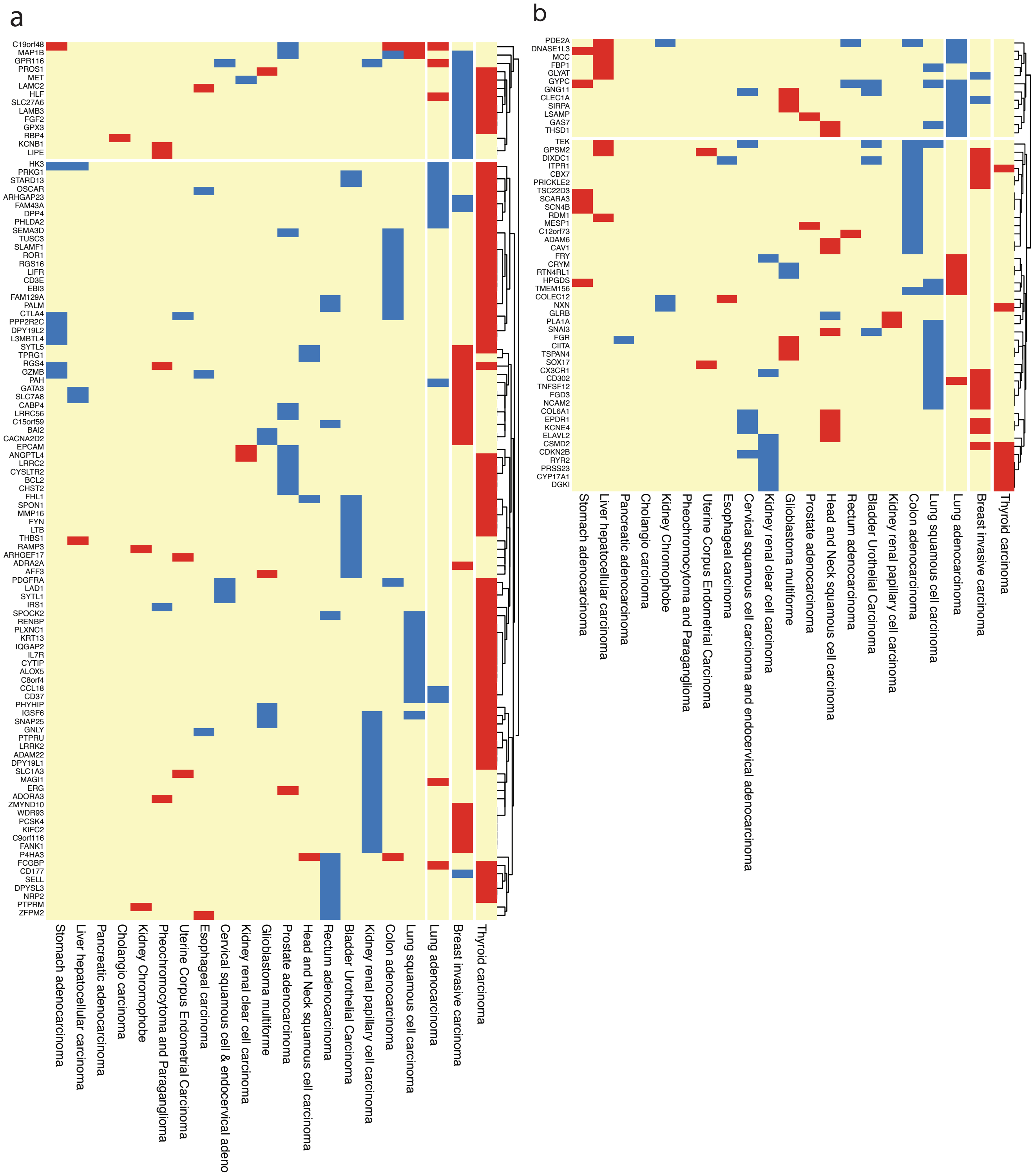

Heatmap showing the 151 dual role genes (rows) within 18 cancer types (columns).

Predicted OCGs (TSGs) are in red (blue).

Supplementary Figure 2

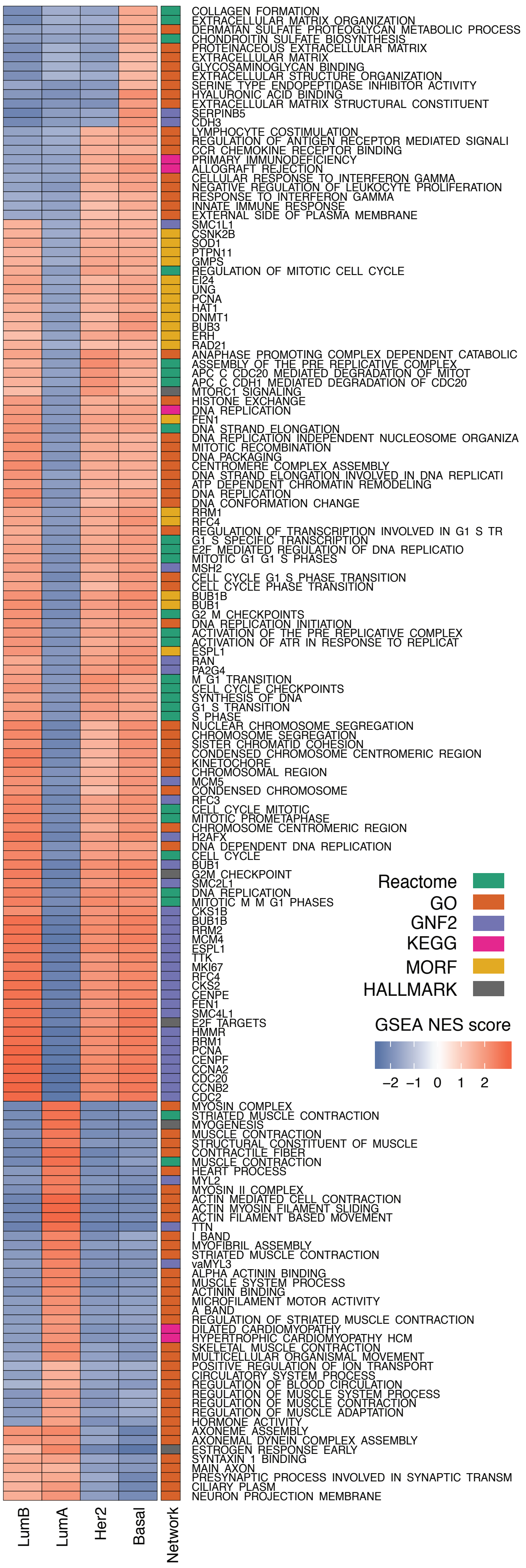

Gene programs associated to Breast cancer molecular subtype detected by Dynamic Recognition Analysis

Supplementary figure 3

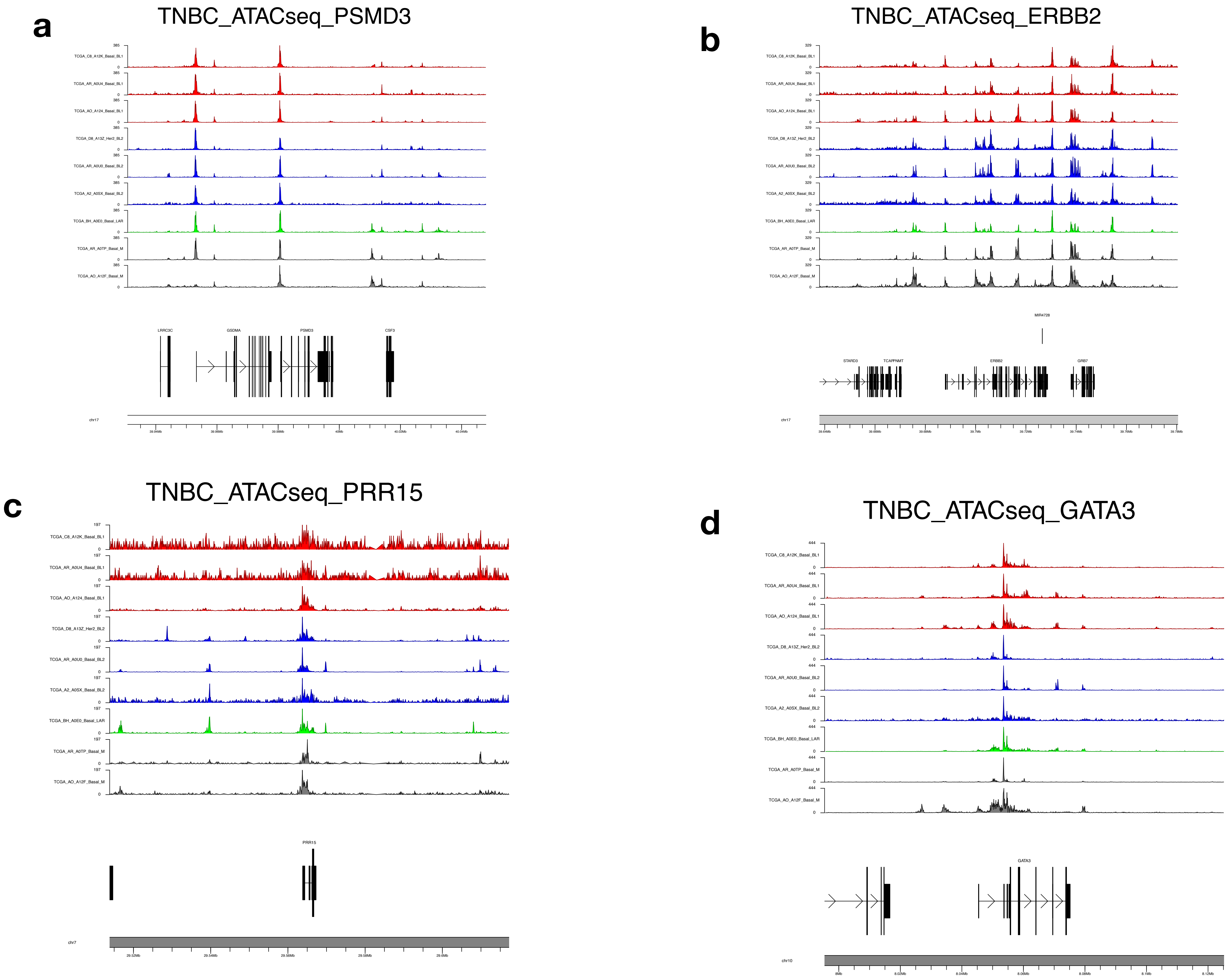

Genome browser screenshots of normalized ATAC-seq sequencing tracks of 13 different breast cancer samples shown across the same (A) PSMD3, (B) ERRB2, (C) PRR15 (D) GATA3 locus. Triple negative breast cancer subtype colored as: BL1 in red, BL2 in blue, LAR in green, M in gray. TCGA BRCA molecular subtypes identified according to criteria described in Methods.

Supplementary Figure 4

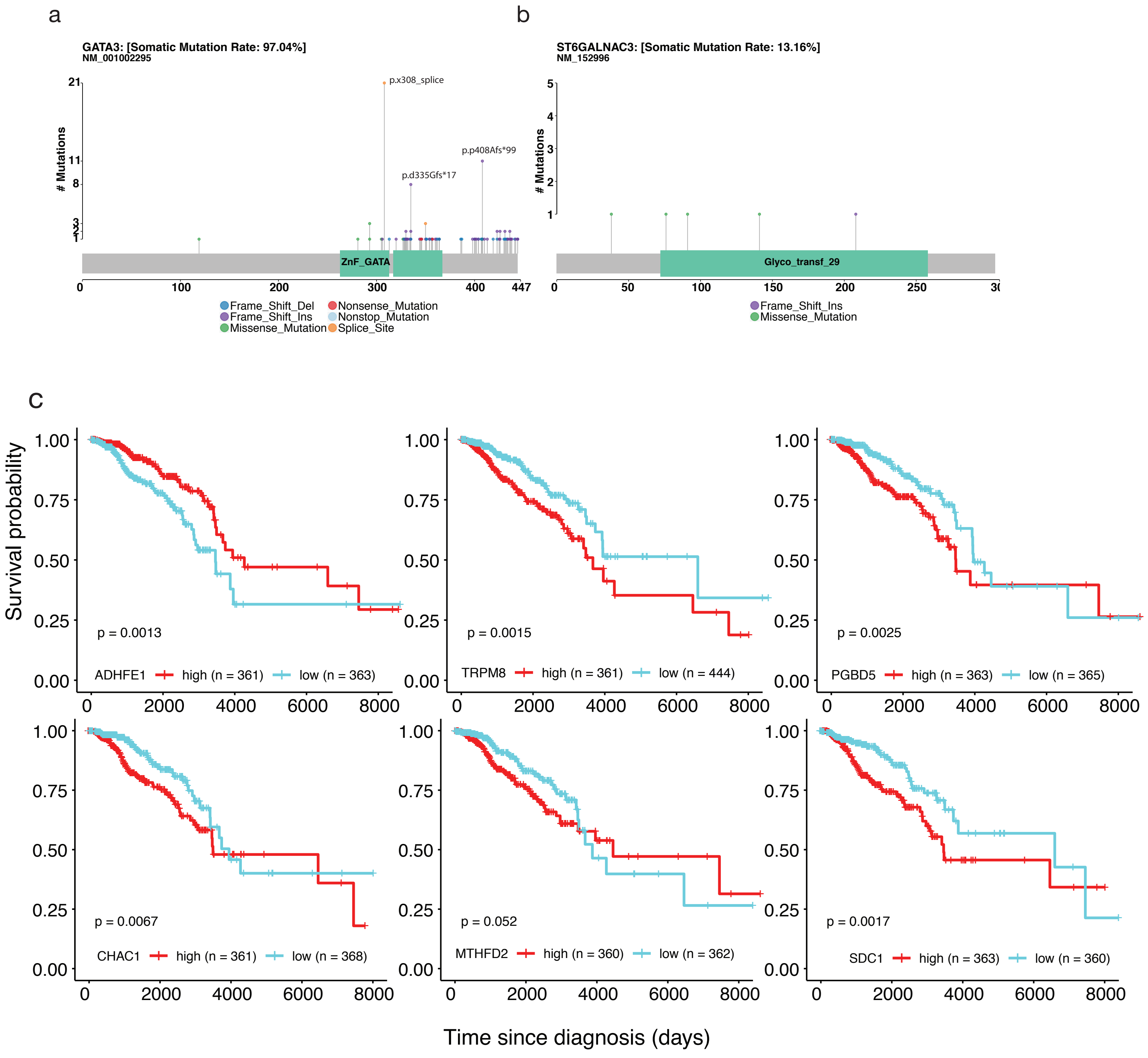

Lolliplot of mutation of GATA3 (a) and ST6GALNAC3 (b) in breast cancer.
(c) Kaplan-Meier Survival curves in TCGA BRCA for OCGs ADHFE1, TRPM8, PGBD5, CACH1, MTHFD2, and SDC1.
