# Supplementary material for: Interpreting pathways to discover cancer driver genes with Moonlight
Source: Nat Commun. 2020 Jan 3;11:69. doi: 10.1038/s41467-019-13803-0 (PMC6941958; doi:10.1038/s41467-019-13803-0)
Supplement: Supplementary file 3 — Description of Additional Supplementary Files [file 41467_2019_13803_MOESM3_ESM.pdf]

Description of Additional Supplementary Files

Supplementary Data 1: 500 Relevant Biological processes from Ingenuity Pathway Analysis (IPA)

Supplementary Data 2: Biological processes selected for Moonlight

Supplementary Data 3: Summary of TCGA RNA-seq samples and Differentially Expressed Genes (Tumor vs normal analysis) in 18 cancer types.

Supplementary Data 4: Functional enrichment Analysis results using the 3390 DEGs between normal and tumor TCGA breast samples

Supplementary Data 5: CDGs for TCGA BRCA molecular subtypes

Supplementary Data 6: Moonlight's oncogenic mediators in 18 cancer types

Supplementary Data 7: TCGA 18 tumor types DMRs overlapping with OCGs and TSG

Supplementary Data 8: Moonlight's oncogenic mediators associated to chromatin accessibility

Supplementary Data 9: Moonlight's oncogenic mediators associated to copy number changes

Supplementary Data 10: Moonlight's oncogenic mediators associated to mutation types

Supplementary Data 11: Moonlight's oncogenic mediators associated with survival outcome in 18 cancer types with good or poor prognosis

Supplementary Data 12: Connectivity MAP analysis of cancer driver genes identified as target of specific compounds

Supplementary Data 13: Moonlight's oncogenic mediators validated by cancer cell lines experiments

Supplementary Data 14: ROMA's results with TCGA BRCA

Supplementary Software 1: Package vignette with R scripts to reproduce the results and figures
