# Supplementary material for: Interpreting pathways to discover cancer driver genes with Moonlight
Source: Nat Commun. 2020 Jan 3;11:69. doi: 10.1038/s41467-019-13803-0 (PMC6941958; doi:10.1038/s41467-019-13803-0)
Supplement: Supplementary file 19 — Reporting Summary [file 41467_2019_13803_MOESM19_ESM.pdf]

nature research

Corresponding author(s): Antonio Colaprico (axc1833@med.miami.edu)

Reporting Summary

Nature Research wishes to improve the reproducibility of the work that we publish. This form provides structure for consistency and transparency in reporting. For further information on Nature Research policies, see Authors & Referees and the Editorial Policy Checklist.

Statistical parameters

When statistical analyses are reported, confirm that the following items are present in the relevant location (e.g. figure legend, table legend, main text, or Methods section).

n/a	Confirmed	
☐	☒	The exact sample size (*n*) for each experimental group/condition, given as a discrete number and unit of measurement
☐	☒	An indication of whether measurements were taken from distinct samples or whether the same sample was measured repeatedly
☒	☐	The statistical test(s) used AND whether they are one- or two-sided<br>*Only common tests should be described solely by name; describe more complex techniques in the Methods section.*
☒	☐	A description of all covariates tested
☐	☒	A description of any assumptions or corrections, such as tests of normality and adjustment for multiple comparisons
☒	☐	A full description of the statistics including central tendency (e.g. means) or other basic estimates (e.g. regression coefficient) AND variation (e.g. standard deviation) or associated estimates of uncertainty (e.g. confidence intervals)
☐	☒	For null hypothesis testing, the test statistic (e.g. *F*, *t*, *r*) with confidence intervals, effect sizes, degrees of freedom and *P* value noted<br>*Give P values as exact values whenever suitable.*
☒	☐	For Bayesian analysis, information on the choice of priors and Markov chain Monte Carlo settings
☒	☐	For hierarchical and complex designs, identification of the appropriate level for tests and full reporting of outcomes
☒	☐	Estimates of effect sizes (e.g. Cohen's *d*, Pearson's *r*), indicating how they were calculated
☒	☐	Clearly defined error bars<br>*State explicitly what error bars represent (e.g. SD, SE, CI)*

Our web collection on statistics for biologists may be useful.

Software and code

Policy information about availability of computer code

Data collection	We used the functions GDCquery(), GDCdownload() and GDCprepare() from the R/Bioconductor package TCGAbiolinks version 2.9.5 (Colaprico et.al, NAR, 2015) to collect the -omics data used in this study (i.e.,gene expression, methylation, clinical and mutation cancer data) in May 2018 from The Cancer Genome Atlas (TCGA) cohort deposited in the Genomic Data Commons (GDC) Data Portal. (https://github.com/BioinformaticsFMRP/TCGAbiolinks)<br>We downloaded the RMA normalized expression data for 1001 Cell lines, from the Genomics of Drug Sensitivity in Cancer (GDSC)'s study in May 2018 using in-house scripts.<br>We also provided the section Code availability in the Methods related to R scripts and software used and deposited in a public available Github repository (https://github.com/ibsquare/MoonlightR).
Data analysis	We used the functions TCGAanalyze(), TCGAvisualize() from the R/Bioconductor package TCGAbiolinks version 2.9.5 (Colaprico et.al, NAR, 2015) to analyze and visualize the results from each analysis related to multi -omics data used in this study (i.e.,gene expression, methylation, clinical and mutation cancer data) from GDC. (https://github.com/BioinformaticsFMRP/TCGAbiolinks)<br>We used Ingenuity Pathway Analysis (IPA) release 2017 to identify 500 relevant biological processes (BPs), we then manually selected 101 BPs known to be relevant in cancer. Afterwards, we used IPA for each gene/ biological process combination, to obtain the number of times (number of publications in PubMed) the pair was mentioned together in terms of upregulated, downregulated or (less specifically) affected expression.<br>We used the Java version 2017 of Representation and quantification Of Module Activity (ROMA)<br>for the pathway activity evaluation (https://github.com/sysbio-curie/Roma).

All statistical analysis were performed using R version 3.5.0 (2018-04-23) software and Rstudio Version 1.1.453.
We used the functions DPA, GRN, FEA, PRA, DRA from the R/Bioconductor package MoonlightR version 1.6.1 (https://github.com/ibsquare/MoonlightR)
We used the Broad Institute's Connectivity Map build 02 (CM) version 2018, a public online tool (https://portals.broadinstitute.org/cmap/) for drug analysis. To further investigate the mechanism of actions (MoA) and drug-target we performed specific analysis within Connectivity Map tools version 2018 (https://clue.io/).
We used the software 20/20+ (version 1.2.0) (https://github.com/KarchinLab/2020plus) and OncodriveRole (version 2018) (https://bbglab.irbbarcelona.org/oncodriverole/) in comparison to the performance of Moonlight.
We also provided the section Code availability in Methods related to R scripts and software used and deposited in Github.

For manuscripts utilizing custom algorithms or software that are central to the research but not yet described in published literature, software must be made available to editors/reviewers upon request. We strongly encourage code deposition in a community repository (e.g. GitHub). See the Nature Research guidelines for submitting code & software for further information.

Data

Policy information about availability of data

All manuscripts must include a data availability statement. This statement should provide the following information, where applicable:

- Accession codes, unique identifiers, or web links for publicly available datasets
- A list of figures that have associated raw data
- A description of any restrictions on data availability

The omics -omics datasets (gene expression, methylation, clinical and mutation) analysed during the current study are public available in the repository (https://portal.gdc.cancer.gov/)
The cell lines dataset analysed during the current study are public available in the repository (https://www.cancerrxgene.org/downloads)
All data generated or analysed during this study are included in this published article (and its supplementary information files and in related publication folder in https://github.com/ibsquare/).

Field-specific reporting

Please select the best fit for your research. If you are not sure, read the appropriate sections before making your selection.

☒ Life sciences ☐ Behavioural & social sciences ☐ Ecological, evolutionary & environmental sciences

For a reference copy of the document with all sections, see nature.com/authors/policies/ReportingSummary-flat.pdf

Life sciences study design

All studies must disclose on these points even when the disclosure is negative.

Sample size	We considered as sufficient a minimum sample size of 5 normal or tumor samples for our RNA-Seq analysis. This number is satisfactory to achieve a power between 0.8 and 1 (one e.g., 100% detection of differentially expressed genes) as mentioned in Ching, et. al, Power analysis and sample size estimation for RNA-Seq differential expression, RNA, 2014
Data exclusions	We excluded cancer type from our pan-cancer analysis that had availability of less than 5 samples of normal tissue of RNA-Seq gene expression GDC.
Replication	We successful verified the reproducibility of our findings, running our R codes / pipelines in several machines with different O.S:<br>Platform: x86_64-apple-darwin15.6.0 (64-bit), Running under: macOS High Sierra 10.13.5<br>Platform: x86_64-pc-linux-gnu (64-bit), Running under: Ubuntu 18.04.1 LTS<br>Platform: x86_64-w64-mingw32/x64 (64-bit) Running under: Windows >= 8 x64 (build 9200)
Randomization	Randomization was not relevant to our study because we didn't collect new samples or generated controlled trials because we used only public available datasets.
Blinding	Blinding was not relevant to our study because we didn't collect new samples or generated controlled trials because we used only public available datasets.

Reporting for specific materials, systems and methods

Materials & experimental systems

n/a	Involved in the study
☒	☐ Unique biological materials
☒	☐ Antibodies
☒	☐ Eukaryotic cell lines
☒	☐ Palaeontology
☒	☐ Animals and other organisms
☒	☐ Human research participants

Methods

n/a	Involved in the study
☒	☐ ChIP-seq
☒	☐ Flow cytometry
☒	☐ MRI-based neuroimaging
